# Mothers’ reproductive and medical history misinformation practices as strategies against healthcare providers’ domination and humiliation in maternal care decision-making interactions: an ethnographic study in Southern Ghana

**DOI:** 10.1186/s12884-018-1916-9

**Published:** 2018-07-03

**Authors:** Linda L. Yevoo, Irene A. Agyepong, Trudie Gerrits, Han van Dijk

**Affiliations:** 1 Sociology of Development and Change Group, Wageningen University, P. O. Box 8130, Hollandsweg 1, 6700 EW Wageningen, Netherlands; 2 Dodowa Health Research Centre, Research & Development Division, Ghana Health Service, P. O. Box DD 1, Dodowa-Accra, Ghana; 3 Graduate School of Social Sciences, Kloveiersburgwal 48 1012 CX Amsterdam, University of Amsterdam, Amsterdam, Netherlands

**Keywords:** Ghana, Care decision-making, Power, Empowerment, Ethnography, Pregnant women, Respectful maternal care

## Abstract

**Background:**

Pregnant women can misinform or withhold their reproductive and medical information from providers when they interact with them during care decision-making interactions, although, the information clients reveal or withhold while seeking care plays a critical role in the quality of care provided. This study explored ‘how’ and ‘why’ pregnant women in Ghana control their past obstetric and reproductive information as they interact with providers at their first antenatal visit, and how this influences providers’ decision-making at the time and in subsequent care encounters.

**Methods:**

This research was a case-study of two public hospitals in southern Ghana, using participant observation, conversations, interviews and focus group discussions with antenatal, delivery, and post-natal clients and providers over a 22-month period. The Ghana Health Service Ethical Review Committee gave ethical approval for the study (Ethical approval number: GHS-ERC: 03/01/12). Data analysis was conducted according to grounded theory.

**Results:**

Many of the women in this study selectively controlled the reproductive, obstetric and social history information they shared with their provider at their first visit. They believed that telling a complete history might cause providers to verbally abuse them and they would be regarded in a negative light. Examples of the information controlled included concealing the actual number of children or self-induced abortions. The women adopted this behaviour as a resistance strategy to mitigate providers’ disrespectful treatment through verbal abuses and questioning women’s practices that contradicted providers’ biomedical ideologies. Secondly, they utilised this strategy to evade public humiliation because of inadequate privacy in the hospitals. The withheld information affected quality of care decision-making and care provision processes and outcomes, since misinformed providers were unaware of particular women’s risk profile.

**Conclusion:**

Many mothers in this study withhold or misinform providers about their obstetric, reproductive and social information as a way to avoid receiving disrespectful maternal care and protect their privacy. Improving provider client relationship skills, empowering clients and providing adequate infrastructure to ensure privacy and confidentiality in hospitals, are critical to the provision of respectful maternal care.

**Electronic supplementary material:**

The online version of this article (10.1186/s12884-018-1916-9) contains supplementary material, which is available to authorized users.

## Background

Dufie, a high school graduate suddenly began to bleed 30 min after delivery of her baby. The healthcare provider re-examining the placenta, saying aloud as she checked over possible causes for the bleeding: *“There is no missing lobe, she had no tear [vagina], and her uterus is well contracted. What is making this woman bleed profusely?”* She massaged Dufie’s lower abdomen gently and instructed the researcher to fetch an ampule of oxytocin injection.

Dufie’s bleeding ceased about an hour later, after receiving nine more ampules of oxytocin injections.^1^ Shortly thereafter, a gentleman came into the labour ward and said: *“My wife telephoned to inform me she had just delivered a baby girl and she is upset about it.”*

The healthcare provider exploded with anger and asked him: *“Papa, are baby girls not human beings? She started bleeding suddenly because she disliked the baby’s sex!”*

The gentleman replied: *“My wife insisted she wanted another male child although we have a son amongst our six children”.* The healthcare provider became angrier at the revelation that a client who had told her she had had only three previous deliveries was actually a grand multiparous woman having her seventh delivery. She asked the gentleman: *“You mean your wife is a mother of seven instead of three? She nearly killed herself because she lied to us in her reproductive history. I would not have managed the bleeding the way I did, if I knew she was a mother of seven.”*^2^

## Reproductive care decision-making interactions: An encounter of domination and resistance

This labour ward scene is one of many observations made of pregnant women’s with-holding vital medical, reproductive and social information from healthcare providers during field work. Withholding of information from healthcare providers by pregnant women (and clients) has also been reported in the literature from other healthcare settings [1–3]. Understanding why this happens in context, is critical for improving care decision-making and health outcomes. This is because the medical, obstetric, gynaecological and social history information pregnant women possess about themselves, is critical for healthcare providers’ care decision-making. Healthcare providers combine clients’ information with their expert knowledge and skills to arrive at what they hope is the appropriate diagnosis and management plan that addresses the client’s condition. Sometimes, with adequate information, healthcare providers may not require further physical examination or laboratory tests to determine the appropriate management clients require [4]. This article aims to provide insight as to why pregnant women in this study misinformed their healthcare providers or withheld specific information during antenatal care; and how this affected healthcare providers’ care decisions and related clinical management and sometimes outcomes. This understanding is essential to inform interventions to improve maternal and newborn healthcare service and quality outcomes.

Researchers on provider-client care decision-making interactions have argued that asymmetrical relations of power between healthcare providers and clients, make healthcare providers dominate the care decision-making process [5]. For instance, healthcare providers may control the way clients -including pregnant women - should interact during the decision-making process, sometimes disregarding their complaints and concerns [6]. Other times, healthcare providers become angry, yell at, verbally abuse and make derogatory remarks about clients when they perceive they are not adopting the ‘appropriate’ medical practices and behaviours [5, 7–11]. In addition, healthcare providers sometimes conduct care interactions in environments that do not take into consideration confidentiality of information clients provide them and their privacy needs [2, 12]. These healthcare provider practices have been claimed to amount to human rights violations and denial of the right of pregnant women to receive respectful and dignified healthcare [13]. Despite this, empirical studies and qualitative synthesis suggest that the problem remains widespread globally, but more prevalent in low and middle-income countries including Ghana, the setting of the current study [8, 14–21]. Some consequences of disrespectful treatment of pregnant women include dissatisfaction, increased risk of experiencing obstetric complications and poor utilisation of reproductive and maternal healthcare services in subsequent pregnancies and deliveries [16, 22–25].

Other writers have suggested that healthcare providers' disrespectful treatment of mothers and practices of power, transforms clients from being independent decision-makers into passive actors during the care consultation [26].

On the contrary, some medical anthropologists and feminist scholars, argue that only analysing healthcare providers’ enactment of (coercive) power during the care decision-making interaction is to overlook how clients respond to healthcare providers’ attempts to dominate them [27–29]. These authors contend that clients including pregnant women do not remain passive. Rather they use whatever resources they possess – e.g., social knowledge about healthcare providers’ attitudes, behaviours etc., − to develop strategies to maintain some control over their situation [30]. Foucault contends, where there is power, there is resistance ([31]: 95-96).

From this later perspective, empirical evidence from the medical literature shows that clients—including pregnant women—sometimes do not fully submit to healthcare providers’ authority. They resist by concealing their medical information from healthcare providers during care decision-making interactions [1, 2]. A study among Latin American pregnant women found that pregnant women concealed some information about themselves from the healthcare providers because they were dissatisfied with the healthcare provider’s relationship with them [32]. Similarly, Lazarus [29] observed that Puerto Rican pregnant women told healthcare providers what they wanted to hear as an expression of dissatisfaction with their healthcare providers behaviour towards them.

Some scholars have attributed similar client ‘resistance’ behaviours to gaps in their medical knowledge which prevent them from sharing vital health information even when given ample opportunity to do so [33].

In this article, we argue that clients’ acts of resistance are directed towards healthcare providers’ behaviours and healthcare delivery practices they perceive as unresponsive and humiliating to their personhood. We draw upon practice theory [34] and anthropological concepts of ‘resistance’, ‘public transcript’, ‘front stage’, ‘hidden transcript’ and ‘back stage’ from the work of James Scott [35, 36] to guide our analysis.

## Practice theory

Practice theory highlights the puzzling and dialectical way(s) less powerful social actors behave in a relationship of domination. They respond by reproducing their own repression, acting in opposing ways or by resisting the submissive behaviour they have been socialised to use towards persons they consider as their superiors [34]. Some analysts contend that subordinated persons have consciousness and ‘see’ through the ‘workings’ of their domination [37] and hence, develop strategies to escape from being dominated [38]. Resistance - also defined as ‘oppositional agency’ [39] - is a strategy that less powerful persons may resort to in order to confront being dominated.

### Resistance

James Scott [36] in his study ‘The Weapons of the Weak: Everyday Forms of Peasant Resistance’, conceptualizes resistance as act(s) that subordinated persons engage in with the intention to deny claims made on them by persons in a superordinate class. Scott argues that the relative powerlessness of subordinated persons, make them use covert resistance strategies (e.g. false compliance) as individualized self-help strategy to express dissent of their domination and confront superiors ([36]: 290). Feminists and scholars of subalterns argue that the strategies of these subordinated persons shed light on how they ‘play with power’ and express agency even under extreme domination ([40]: 12). In this article, we contend that because subordinates are not a homogeneous group, how they decide to act in power-laden relationships may vary depending on how they anticipate more powerful actors will (re)act towards them and on their interpretations of the dominator’s behaviour.

### ‘Public transcript’, ‘front stage’, ‘hidden transcript’ and ‘backstage’

‘Public transcript’ is a resistance strategy that subordinates use to evade domination and abuse of powerful persons’ during public interactions [35]. ‘Public transcript’ consists of deliberately misrepresenting oneself by ‘tilting’ one’s discourses (speech, emotions etc.) to conform to powerful persons’ expectations on the ‘front stage’ [35]. The ‘front stage’, is a social space bounded by precepts of ‘appropriate’ conduct ([41]: 107), and a site where power relations are manifested and made visible. The ‘front stage’ is set to make subordinates act according to the ‘rules’ of conduct because subordinates’ socialization tells them the way to behave on the ‘front stage’; and to anticipate possible reactions of their superiors when they [subordinates] speak their mind or do not act according the ‘rules’ of the ‘front stage’ [35]. Therefore, on the ‘front stage’, subordinates’ speak the lines of their superiors as a strategy to reassure their superiors they are in ‘control’ ([35]: xii). The accommodative attitude of the subordinates makes it difficult for their superiors to determine where subordinates’ compliance ends and resistance begins ([36]: 289).

As a result, the way subordinates act in the presence of their superiors can diverge from their behaviours ‘backstage’. Discovering what subordinates ‘actually’ feel, say and do, requires exploring their ‘hidden transcript’ [35]. ‘Hidden transcript’ to a large extent are discourses and practices that subordinates engage in whilst they are ‘backstage’ out of sight and earshot of superiors ([35]: 4). ‘Backstage’ is a ‘social space’ where subordinates interact with persons they ‘trust’ and take off the guises they put up whilst in the presence of their superiors [36]. These ‘backstage’ acts and ‘hidden transcript’, represent subordinates’ agency. The ‘front’ and ‘back stages’ also include subordinates’ behaviour surrounding concerns, anger, dissatisfaction and criticism of their superiors. In our work, uncovering pregnant women’s ‘hidden transcript’ was essential to understanding their behaviour when interacting with healthcare providers in the antenatal care units and maternity wards in our study setting in southern Ghana.

## Methods

### Setting

Though Ghana’s infant and maternal mortality have been falling over time, they remain unacceptably high. The maternal mortality ratio is estimated to have fallen from 634 per 100,000 livebirths in 1990 to 319 in 2015 [42] and neonatal mortality rates have declined from 41/1000 live births (1983–87) to 29/1000 live births (2010 to 2014) [43]. The Greater Accra Region where the study was conducted was one of the first sites in Ghana where institutionalisation of childbirth began [44, 45]. It currently holds the country’s capital, the seat of government; and has relatively better roads and healthcare infrastructure than other regions in Ghana. These amenities make it a preferred region for healthcare professionals, contributing to the continuing geographical maldistribution of the country’s critical healthcare professionals for maternal and newborn care [46]. Despite its relatively privileged status in the Ghanaian context, the region’s institutional maternal mortality ratios were recorded to have worsened since 2011 [47, 48]. We conducted our study in two hospitals in this region which, for reasons of confidentiality we have named the Moon and Dawn hospitals. The Moon hospital was the smaller of the two study hospitals and the Dawn Hospital was relative better resourced in terms of healthcare infrastructure compared to the Moon Hospital. However, in absolute terms, both hospitals were resource and infrastructure constrained with severely limited operational working space and privacy for antenatal care and clinical consultations.^3^^,^^4^

### Study design and data collection

This work was part of a larger ethnographic study undertaken in these two public hospitals to gain an in-depth understanding of care decision-making processes for mothers and newborn, and factors that influenced this process. The ultimate aim was to generate empirical evidence to inform design and implementation of interventions to accelerate improvements in maternal and new born health outcomes. The first author, henceforth, referred as the ‘researcher’, conducted ethnographic fieldwork to explore interactions between healthcare providers of maternal and newborn services and pregnant and delivering women in the antenatal, postnatal departments and labour wards in the two hospitals. The researcher spent a total of 22 months in the two hospitals in periods between a month and six months at a time between — February 2012 and July 2014— with participant observation used as the main approach to data collection.

Participant observation is the process whereby the researcher finds a credible role to facilitate the researcher’s participation in the daily lives of research informants in their ‘natural’ setting [49]. This helps the researcher to participate, observe and create rapport between the researcher and the research informants [50]. In addition, it enables the researcher engage in conversations and seek clarification from informants about their behaviour and the meanings they attached to what they do. This process helps the researcher to understand what informants do within the context of their acts [51]. The in-depth interactions and observations are captured and written as field notes on daily basis. In the hospital settings the extent of the researcher’s participation in informants’ daily lives can sometimes be problematic for ethical and practical reasons like the researcher’s personal identity and the subject of interest [49].

In this study, the researcher used her student researcher identity and the permission given by the hospital management, to participate in and observe care giving process and interactions between healthcare providers and pregnant women to answer the research questions. Using the student researcher identity enabled her to be present in the antenatal care units and the labour wards of the two hospitals. To prevent the researcher from being a nuisance to healthcare providers [52], and also play a meaningful role in the daily lives of healthcare providers and pregnant women, the researcher was ‘informally’ trained by healthcare providers to capture data about pregnant women. In the antenatal care unit she assisted the healthcare providers with recording data on mothers’ socio-demographic and obstetric information, maternal weight and height. In the labour ward, she assisted with recording data such as date and time of delivery of the baby and placenta as well as the height and weight, chest and head circumferences of the baby. These are tasks healthcare providers also train female cleaners and student nurses to perform. During data capture in the antenatal care unit the researcher developed the habit of investing a little time to inform pregnant women about the importance of the accuracy of information for receiving quality care. She also took pregnant women a little away from the more public environment where the mother’s histories were taken and invested time in answering women’s questions repeatedly without showing signs of impatience and irritation. The practice she adopted made the history taking process longer but was possible because she had more time to spare as a student researcher. It also allowed her to gain the women’s ‘trust’. As this happened they started to tell her about their plans to limit the provision of some obstetric information and sometimes asked the researcher to correct information they had earlier misrepresented.^5^^,^^6^^,^^7^^,^^8^

This initial observation led to the researcher deciding to focus more time within her overall study on understanding what was happening in the area of histories provided by pregnant women at the antenatal clinic and why it was happening. She therefore focused on obtaining further insights from a sample of pregnant women on the range of information they withheld or misrepresented about themselves during history taking and observing care decision-making interactions between them and healthcare providers of over a longer period of time. In order to make these focused observations of the antenatal care history taking process, a total of 81 pregnant women who were within a gestational age of between 12 and 20 weeks were purposively selected and invited to participate in a longitudinal follow up.

The selected women were informed about the study’s objective and data collection processes. Forty-two of these pregnant women - 24 in the Dawn and 18 in the Moon hospital -  gave verbal consent to be followed-up when they reported on scheduled antenatal care appointments. Of the thirty-nine (39) women who refused to participate in the study, 15 of them needed spousal consent, 16 stated no reasons for their refusal and eight of them said they were considering the invitation but did not give a reply to the researcher, even though they subsequently often came into contact with the researcher. Given the context of ‘mistrust’ of healthcare providers, it is possible that the women who refused to participate were more inclined to keep their ‘hidden transcript’ hidden or were less trusting of the researcher’s intent.

Upon acceptance, contact numbers were exchanged between the researcher and the pregnant women, and their socio-demographic information were documented. The exchange was to develop and facilitate long-term relationship and interactions between the researcher and the pregnant women. At pregnant women’s antenatal care appointments, the researcher sat in the consultation process to observe the care interaction with healthcare providers, through several antenatal care appointments. Initially, the pregnant women seemed uncomfortable with the researcher’s presence in the consultation process. Over time this changed, because at subsequent antenatal care appointments, pregnant women prompted her to come along when it was their turn to consult with healthcare providers. Brief field notes of interactions between healthcare provider and women were written and later expanded in the evenings in line with ethnographic standards and to aid analysis [53].

Subsequently, the researcher interviewed pregnant women to seek information they misrepresented about themselves or withheld from healthcare providers during history taking and in the care decision-making interactions and reasons that explained their actions. She used a semi-structured observation and interview guide (Additional file 1). At the end of the fieldwork period, the researcher had consistently observed all care decision-making interactions of 18 women at the Dawn Hospital from the first antenatal care registration until the postpartum period. For several practical reasons, the researcher was not able to consistently follow-up all the 24 women in the Moon hospital.^9^

To validate information generated from observation of the selected pregnant women in care decision-making interactions, and to verify whether the misinformation and withholding of information practices of pregnant women at the antenatal care was the norm rather than an exception, four focus group discussions were held with pregnant and postnatal women using a focus group discussion guide (Additional file 2). Three groups consisted of a mix of women with different levels of education and one consisted of women with a secondary education or higher. Two reasons accounted for the extra separate focus group discussion with the more highly educated pregnant women. First, women with a low level of education formed the majority of the hospitals’ clientele and also made up the majority of pregnant women who participated in the study. Education and literacy can empower women and potentially reduce the power distance between healthcare providers and clients. It was therefore useful to find out practices and views between relatively highly educated (completed high school or more) and less highly educated women. The participants included women who had received prenatal, delivery and postnatal care from one of the two hospitals and women who attended antenatal care in other facilities (e.g., private or community clinics) but accessed skilled delivery care in the study hospitals because their prenatal clinics did not offer any skilled delivery services or they were high-risk cases referred for specialised obstetric care. Each focus group discussion was made up of between 7 and 10 women and was held with women ‘backstage’. ‘Backstage’ [35] in this context was a rented premise located away from the hospital and healthcare providers. It provided the women with an environment to feel at ease and freely discuss their behaviours and experiences with the research team. Also, during the focus group discussions, women were asked not mention their names or the names of healthcare providers and to replace the names of their hospitals with the pseudonym ‘my hospital’ when making contributions. It was for anonymity reasons and to encourage them discuss issues freely.

A research assistant with experience in qualitative data collection including moderating focus group discussions was recruited as the facilitator for the discussions and the researcher took hand-written notes. The researcher paid the women’s transportation costs to and from the venue and provided snacks during the discussions. The discussions lasted approximately 90 min each and were held in a local language (Twi) and audiotaped. Women’s responses were transcribed verbatim by an independent research assistant for validity purposes; and were translated into English in the process.

### Data analysis

Data collection and analysis was an iterative process according to the canons of grounded theory [54]. The data generated from the multiple sources were read line-by-line, and were sorted, integrated, organized and open-coded manually. The texts were categorised into themes and subthemes and given analytical labels. These categories were compared and contrasted between the various methods according to women’s educational level and hospitals. The product of the process is presented as a ‘thick description’ [55] in the following sections.

## Results

### Managing obstetric and maternal information to resist healthcare providers’ ideological ‘domination’ and humiliation

The researcher asked Dufie described in the vignette at the beginning of this paper, why she had not disclosed the number of children she had had during her history taking. She responded that her personal experiences with and observations of healthcare providers’ relationships with multiparous pregnant women were often negative. She anticipated that the healthcare provider would have similar negative reaction towards her information about being a mother of six children and pregnant with a seventh. She said, the healthcare provider’s likely comments to the ‘right’ information would be: “Why are you are having too many children? In contemporary Ghana with so much economic hardship; you are not practicing family planning.” She went on to say:

*“I make the decision to have ten children or less. Therefore, in my interactions with the healthcare provider, I expect her to talk to me nicely and explain why I should have fewer children rather than insulting me into submission. […] you tell her what she wants to hear [...]. After all, they cannot follow me to my house to physically count the actual number of children I have.”* 10

Dufie’s position was confirmed by many of the pregnant and postpartum women who participated in the focus group discussions. The women interpreted healthcare providers’ reactions as attempts to control them to think and act in ways that conformed to healthcare providers’ reproductive ideologies and values. According to them, healthcare providers were ignoring the fact that they are autonomous individuals with the right to make their own fertility choices.^11^^,^^12^

Many of the women expressed concerns about healthcare providers’ negative behaviour towards them such as making derogatory comments and criticising them when the obstetric information they gave did not conform to healthcare providers’ reproductive ideologies. They explained in the focus group discussions and interviews that telling healthcare providers what they want to hear, prevented healthcare providers’ verbal insults and also prevented them [women] from getting angry and starting arguments with the healthcare provider because of the insults.^11,12,^^13^^,^^14^^,^^15^

Pregnant women who sought antenatal care late in pregnancy - especially in the third trimester of pregnancy - sometimes reduced their gestational age to prevent healthcare providers from chastising them because they had failed to seek early antenatal care. Abigail a mother with no formal education attended her first antenatal care at a late gestational age. Though her ultrasound scan result indicated a higher gestational age, she told the attending healthcare provider a lower pregnancy gestational age. It was because she feared to be chastised by the healthcare provider if she told the truth of her real gestational age, which was too late for a first antenatal care visit. The healthcare provider found out her real gestational age through the ultrasound scan results and Abigail’s fears of chastisement proved right. A part of the transcript of her interaction with the attendant healthcare provider shows below:

*Healthcare provider: “Your scan result suggests you are almost due for delivery, but this is your first antenatal care visit. [...]. Pregnant women are stubborn. I am sure you are ill, the reason you are here today. Do you feel unwell?”*

*Abigail smiled at the healthcare provider and said: “I have leg pains and a headache”.* 16

Despite being verbally abused, Abigail kept her fake smile. She told the researcher later: *“The healthcare provider’s behaviour did not bother me because they always want to disgrace us.”* 17

Women in the focus group discussions confirmed that many pregnant women like Abigail gave a lower gestational age when they were registering for antenatal care in the third trimester because they feared healthcare providers would verbally assault and humiliate them.^11,12,13^

Rhoda, a highly educated mother criticised healthcare providers’ for this behaviour during the group discussion. She said:

*“If you start antenatal care around six months, these healthcare providers will get angry and treat you in an inhuman way. They make sure to stand facing the crowd of pregnant women to criticize and insult you. If arriving for antenatal care later warrants such disgrace, when they [healthcare providers] ask you the pregnancy gestation, you reduce it by maybe three months. When the repercussions begin to manifest, we will both suffer because they would be running helter-skelter to save me.”* ^12^

Women further explained that they often kept quiet when healthcare providers became angry, because engaging in open confrontation with them meant one may have to reluctantly terminate seeking care in the facility and move to another, located further away from your place of residence. Moving to another facility, the women said had negative consequences like incurring additional economic and social costs, such as increased transportation and time costs and social inconvenience. They also felt that healthcare providers were likely to neglect them for perceived act of insubordination if they openly confronted them.^12^^,^^13^^,^^17^^,^^18^

The women also indicated that some pregnant women - especially older women - sometimes altered information about their age to avoid being chastised and humiliated by the healthcare providers. Mothers revealed in the focus group discussions that they knew some older pregnant women – generally 40 years or older - who altered their age by at least ten years during history taking to prevent care provider reprimands. Susana, an uneducated mother observed that healthcare providers often berated these older women and made them feel like criminals for carrying a pregnancy at an older age. She stated:

*“Many reasons account for women’s decision to get pregnant at an older age. [...]. But these healthcare providers will humiliate her and associate the pregnancy with the refusal to adopt family planning. Generally, no one wants to be treated like a criminal when seeking care. [...] so under such situation you will reduce your age if asked.”* 19 ^,^ 20

Comments made by some healthcare providers’ in the study suggested that the women’s concern about being chastised for “incorrect” behaviour was not misplaced. The healthcare providers also had motivations and pressures driving their behaviour. One healthcare provider stated in a conversation:

*“Our less educated women do not understand that the simple use of family planning can reduce their risk of maternal morbidity and mortality. They think giving birth so many times is a reward.”* 21

Another healthcare provider said a reason underlying their behaviour was the need to achieve the health sector’s goals for mothers and babies. The healthcare provider stated:

*“There is pressure on us to meet the country’s health sector’s goal of achieving zero maternal death and minimising neonatal mortalities. So you become frustrated that women cannot obey simple instructions or adopt practices that can save their lives and that of babies. That is why we get angry especially at the obstetric information”* 22

### Managing medical information to evade stigmatisation and discrimination

A few of the women in the focus group discussions mentioned that they knew of pregnant women who had withheld vital medical information from healthcare providers because they feared stigmatisation and discrimination. Mandy, a highly educated pregnant woman said:

*Many pregnant women who have HIV or a stigmatised disease like cancer would never disclose it to the doctors [healthcare providers] unless they test them. They do not provide them this information because if they [healthcare providers] get to know that one has HIV, they would pretend they care about her like any other woman. But some behaviour they put up when giving care, let you know that they are not treating her like a human being. For example, they would attend to her last and shout at her, not to touch them.* ^14^

Data from observations of skilled delivery care revealed that healthcare providers did indeed worry about being touched by pregnant women because of the healthcare providers’ concerns and anxiety about occupational exposure to infections. This healthcare providers’ behaviour created a sense that they visibly discriminated against some pregnant women because the adherence to standard infection prevention precautions were not consistently and routinely enforced in the same way for all pregnant women. For example, if a woman was known to be HIV positive, healthcare providers might wear extra pairs of examination gloves in addition to elbow length sterile gloves and any protective clothing they could lay their hands on. Also, they might caution colleagues who were unaware of the pregnant woman’s HIV positive status to protect themselves by winking at them and mentioning a special HIV-positive code, while referencing the mother.^23^^,^^24^^,^^25^^,^^26^

Not all pregnant women responded to dominating and humiliating experiences by withholding information from healthcare providers. Women, who had had previous obstetric complications or had on-going complications particularly, used strategic perseverance as a counter-strategy against provider domination. An example was an uneducated mother, Samantha. She told the researcher that fear of the healthcare providers’ wrath is a reason many expectant mothers withhold information from them during the care decision-making interaction. However, personally, she thought it was still necessary for pregnant women to provide accurate information to their healthcare providers to receive quality care. She explained how her decision to provide the healthcare providers with the required information, had helped her to overcome a threat to her pregnancy:

*“I experienced complications at the early stages of this current pregnancy, so I told the doctors [healthcare providers] all information they required and it has helped me because they gave me the right management, now my condition has improved.”* 27

In another example Patience, a high school graduate mother, recounted her experience in the focus group discussions:

*“Not every one of them [healthcare providers] behaves towards us in a negative manner. In my previous pregnancy, the healthcare provider I met on my first care encounter in this hospital said one of the pregnant women had annoyed [her] so she refused to listen to [a] health complaint from the rest of us. I returned to the hospital again because I often encounter a lot of complications in my pregnancies. This time I met another healthcare provider, who was welcoming. I gave the healthcare provider all my past obstetric information. Immediately I was referred to see the appropriate healthcare provider for my health condition.”* ^12^

### Privacy and confidentiality

The environment in which pregnant women’s reproductive history was taken did not offer privacy because of infrastructure and space constraints and related overcrowding. Healthcare providers took histories without a screen separating the other women waiting in both hospitals. ^4^^,^^5^

The history-taking environment transformed a private interaction between client and healthcare provider into a ‘public conversation’ which others could listen to. Women considered providing certain histories such as induced abortions in such a ‘public’ history taking environment humiliating. Genevieve, a highly educated mother said:

*“People call you a bad girl [promiscuous person] if you have had more than one self-induced abortion. However, as part of the information gathering, the healthcare provider will ask how many self-abortions you have caused whilst other people listen. That is the reason pregnant women often reduce the figures in this context.”* ^17^^,^^22^^,^ 28 ^,^ 29

At the Dawn Hospital, two doctors shared one consulting room and engaged in care decision interactions with pregnant women at the same time.^13,^^30^^,^^31^

Many of the mothers also expressed concerns about healthcare providers’ inability to give them assurances about the protection of the confidentiality of the information they exchanged with them.^12,17,^^32^^,^^33^ This prevented mothers from opening up completely because they were uncertain where their health and reproductive information could end up. The women felt that sharing confidential information could have negative repercussions on their relationship with family members, especially husbands. Ama, a high school graduate, complained:

*“[The] healthcare providers are often unable to speak with clients [in an] undertone. While you give them information, they repeat it shouting because the hospital is noisy. As soon as this happens other patients’ attention is drawn and then they start listening into the interaction. Thereafter, you hide the rest of the information.”* 34 ^,^ 35

## Discussion

Findings from this study corroborate the findings of some other studies [2, 19, 29, 56]. The unequal power relations between healthcare providers and women due in part to the biomedical knowledge healthcare providers have, which they consider as more ‘authoritative’ than that of pregnant women [7, 57]. Additionally, pregnant women desire to receive medical treatment and have access to medical resources like medications, which healthcare providers control [19, 29, 58]. Relatively lower educational and social status of many pregnant women [19, 21, 25, 59, 60] have also been stated as factors influencing pregnant women’s decision to use covert ‘resistance’ strategies to confront healthcare providers, when dissatisfied with their – healthcare providers’ - behaviours and practices of power.

While agreeing with these authors, we think that other practical and socio-cultural reasons may also account for the decision of pregnant women in this study to adopt these covert strategies to resist their domination. Some of the pregnant women in this study were secondary school graduates or even more highly educated than some of the healthcare providers. Despite this, they rarely openly confronted healthcare providers when they were upset with their dominating attitudes and practices of power. How can we understand these findings?

Firstly, it is possible that the general macro socio-cultural context of a fairly hierarchical society and a tendency towards unquestioned deference to authority and powerful persons - whether based on age, social position and control of particular resources [61] - may be influencing the micro social context in the health care seeking and provision interactions. Socially, persons perceived as subordinates would consider it unacceptable to either openly challenge their ‘superiors’ or question their authority, regardless of what they think. This social norm can make it possible for persons who find themselves in a superordinate position in any social, economic or bureaucratic organization to treat and relate to clients regarded as subordinate with disrespect and perceive them as not having a voice to negotiate for services based on their preferences. People, who find themselves in powerful positions, can frustrate less powerful persons who criticise them for perceived abuse of power, and may decide not to provide them with the required service. Some of the pregnant women expressed fears that they might be frustrated or ‘punished’ by the healthcare providers if they openly confronted them for perceived abuses of power. This concern could partially explain why they adopted ‘public transcript’ and ‘front stage’ behaviours. This finding would be keeping with McMahon et al’s [19] study, which suggested that the fear of healthcare providers’ neglect was a reason mothers in Tanzania resigned themselves to endure it, when they were verbally or physically abused by their healthcare provider during childbirth.

The pregnant women in our study - in their pragmatism [40] - evaded open conflict and resorted to more subtle strategies like ‘public transcript’ to respond to healthcare providers whose behaviour dominated, humiliated or stigmatised them.

Related to macro context factors is the general social pressure on individuals to be perceived as respectful [62] and good persons [63] rather than acquiring negative labels such as ‘insolent’ and ‘trouble maker’. These socio-cultural values and pressures may also have prevented the pregnant women in the study from speaking up about particular social interactions and relationships of domination. Adherence to these dominant social values can make women suppress their anger by adopting ‘front stage’ behaviours like maintaining a false smile (as Abigail did). Ghanaian healthcare providers are embedded in the same socio-cultural context where these pregnant women operate, therefore, the way they act and interact with clients may be seen as a microcosm of what pertains in the Ghanaian socio-cultural context (cf. [64]), in relation to customer-patron relationship. Furthermore, healthcare providers often fail to acknowledge that pregnant women are part of a patriarchal system that compels them to have too many pregnancies and denies them the basic right to make their own reproductive decisions [65].

In this article our emphasis – based on a grounded analysis of the research findings – is on an analysis of the reasons that make pregnant women withhold information from health care providers. However, the research also provided insight in the healthcare providers’ behaviour. Some of the healthcare providers in this study mentioned that part of their behaviour is influenced by the pressures on them from their superordinates’ in the healthcare system to reduce maternal and neonatal mortalities to the barest minimum and meet targets like the millennium and sustainable development goals in a resource constrained context. Another study from Ghana describes healthcare providers’ physical abuse of mothers in labour as a strategy for gaining mothers’ cooperation and compliance to improve neonatal outcomes [10].

Another explanation for some of our observations lies in street level bureaucracy theory. The study context is one of over stretched resources, overcrowding, heavy workloads and demand generally outstripping supply. Choices can also be limited, as clients like these pregnant women are “non-voluntary” ([66]: 54). They may not have ready access to private healthcare facilities in their localities and where they were available; the pregnant women may not be able to afford their relatively high costs. Also, clients cannot discipline healthcare providers and healthcare providers have little to lose, even when pregnant women would terminate their relations with their hospitals due to dissatisfaction with a healthcare provider’s behaviour. Some research studies have documented healthcare providers putting quotas on the number of patients they can attend to in a day or sending clients away to minimise workload and reduce stress [9, 11, 67].

Healthcare providers behaviours may also be driven by motivational issues related to organizational conditions and processes such as poorly resourced healthcare facilities, overworked healthcare providers as well as superiors disrespectful treatment of subordinates by being quick to reproach them for mistakes committed while failing to provide equally swift commendation for good work done [24, 59, 67, 68]. Perceptions that employers and managers do not care or give adequate attention to their personal protection or healthcare needs in the face of the risk of injury in line of duty [67], could be part of the reasons for the described subtly stigmatisation and discrimination against HIV positive pregnant women.

As part of interventions to improve maternal and newborn care and outcomes, priority must be given to improving healthcare providers’ client interactional skills by introducing and teaching it at both the basic and continuous learning levels. The skills acquisition may make healthcare providers more sensitive and aware of clients’ aspirations and preferences during care interactions. The responsiveness may have positive effects on trust relations between pregnant women and healthcare providers, and consequently minimise pregnant women’s resistance acts against healthcare providers like withholding aspects of reproductive and medical information.

Healthcare providers’ education should also include information on the importance of patients’ autonomy, informed choices and shared-decision making.

Simultaneously, empowering pregnant women through education on interventions such as the patients’ charter and patients’ rights so that they are better able to speak up when dissatisfied with healthcare providers’ attitude is important. Inventions like creating visible grievance centres in hospitals and a toll free customer hotlines to address clients concerns and prompt investigation to address them should be considered.

Facilitative supervision of healthcare providers to observe how they interact and relate to pregnant women during antenatal care history taking and care decision-making, can assist supervisors to identify peculiar challenges and concerns healthcare providers have other than interactional skills that promote disrespectful treatment of pregnant women. Addressing identified challenges and concerns, and correcting healthcare providers in a blame free manner, may minimise the negative behaviours towards pregnant women. Hospital managers should also recognise and reward staff who provide patient-centred care to minimise disrespectful care practices.

Healthcare administrators also need to pay attention to their responsiveness towards healthcare providers. This includes dealing with safety in the work place, adequate infrastructure and paying attention to work load and stress management. Ensuring the safety and healthcare needs of healthcare providers can improve health worker motivation and consequently may have positive influence on their relationship and attitudes towards clients. Finally, improving privacy and confidentiality in hospitals through provision of adequate infrastructure is critical, and should be seen as part of interventions for safe motherhood.

## Limitations and strengths

The major limitation of the study is that it is focused on in-depth exploration and understanding of issues rather than statistical generalizability. It therefore cannot be assumed that the findings are generalizable beyond the two hospitals. The large number of invited women who refused to participate in the longitudinal observational study of their care interactions with providers means that it is possible that we missed some other unique perspectives peculiar to the pregnant women who refused to participate. There is the possibility that, in the same way that the pregnant woman withheld information from healthcare providers, they may have also hidden some information from the researcher. The use of focus group discussions with women away from the hospital to supplement the direct observational as well as triangulating the responses and observations from focus group discussions, direct observations, interviews and conversations are all strategies that will reduce these possible errors in the data. The strength the methodology is that, it has provided valuable in-depth insights that could not have been uncovered by more statistically generalizable research methods like surveys.

## Conclusion

In developing interventions to improve maternal and newborn health outcomes, much attention is paid to technical quality of care. Less attention has been paid to issues of responsiveness such as the quality of communication between healthcare providers and pregnant women during health care interactions and its implication for quality care provision. As our study shows, poor provider responsiveness can affect information provision by clients, which in turn can affect the quality of clinical decision-making and ultimately maternal and newborn health outcomes. The postpartum haemorrhage scene we described to start this article was in effect a near-miss influenced in part by the withholding of information from the healthcare provider.

## Additional files

Additional file 1: Semi-structured observation and conversation guide. Guide used to capture observations and interactions between pregnant women and healthcare providers during care provision. To also capture interactions between healthcare providers concerning mothers’ care and interview with pregnant women. (DOC 80 kb)

Additional file 2: Focus Group Discussion Guide (FGD guide). A topic guide used to explore in-depth pregnant women reproductive and medical misinformation practices among pregnant women and postnatal mothers. (DOC 64 kb)

## Abbreviations

ANC: Antenatal care
FGD: Focus group discussions
HIV: Human Immunodeficiency Virus
MDG: Millennium Development Goal

## Footnotes

1 This insight also refers to the technical skills of the provider and organisational culture related issues. There is not the focus of this current article as they are addressed in another manuscript.

2 Field notes observation

3 Field notes observation

4 Field notes observation

6 Field notes observation

7 The effect of the researcher’s attitude and presence in the field on the data generated is discussed elsewhere

8 Clients statements to the researcher such as: “You are different from what we know about most ‘healthcare providers’. I had wanted to withhold some reproductive information to avoid the health care provider’s scolding if I told the truth.”

9 In three cases the researcher was engaged in other school-related activities, three pregnant women had their babies closer to their parents; five did not attend their antenatal care appointment regularly. Another three of the women did not want the researcher’s present at their baby’s delivery. They cited embarrassments as reasons and five went into labour at times impossible for the researcher to be present

10 Conversation

11 FGD

12 FGD

13 FGD

14 FGD

15 Interview

16 Field notes observation

17 Conversation

18 Conversation

19 Conversation

20 Conversation

21 conversation

22 Conversation

23 Field notes observation

24 Field notes observation

25 Field notes observation

26 Field notes observation

27 Interview

5 Conversation

28 Field notes observation

29 Field notes observation

30 Interview

31 Field notes observation

32 Interview

33 Field notes observation

34 Conversation

35 Interview

## References

[1] Combs Thorsen V, Sundby J, Malata A (2012). Piecing together the maternal death puzzle through narratives: the three delays model revisited. PLoS One.

[2] Yakong V, Rush K, Bassett-Smith J, Bottorff J, Robinson C (2010). Women’s experiences of seeking reproductive health care in rural Ghana: challenges for maternal health service utilization. J Adv Nurs.

[3] Senah K, van der Geest S, Reis R (2002). Doctor-talk’ and patient-talk’. Power and ethnocentricism in Ghana. Ethnocentricism: Reflections on Medical Anthropology.

[4] Hampton J, Harrison M, Mitchell J, Prichard J, Seymour C (1975). Relative contributions of history-taking, physical examination, and laboratory investigation to diagnosis and management of medical outpatients. Br Med J.

[5] Maternowksa M, Russell A, Sobo E, Thompson M (2000). A clinic in conflict: a political economy case study of family planning in Haiti. Contraception across cultures: technologies, choices, constraints.

[6] Rogers M, Todd C (2002). Information exchange in oncology outpatient clinics: source, valence and uncertainty. Psycho-Oncology.

[7] Jewkes R, Abrahams N, Mvo Z (1998). Why do nurses abuse patients? Reflections from south African obstetric services. Soc Sci Med.

[8] D’Ambruoso L, Abbey M, Hussein J (2005). Please understand when I cry out in pain: women’s accounts of maternity services during labour and delivery in Ghana. BMC Public Health.

[9] Bohren M, Vogel J, Tunçalp Ö, Fawole B, Titiloye M, Olutayo A, Ogunlade M, Oyeniran A, Osunsan O, Metiboba L (2017). Mistreatment of women during childbirth in Abuja, Nigeria: a qualitative study on perceptions and experiences of women and healthcare providers. Reprod Health.

[10] Rominski S, Lori J, Nakua E, Dzomeku V, Moyer C (2016). When the baby remains there for a long time, it is going to die so you have to hit her small for the baby to come out: justification of disrespectful and abusive care during childbirth among midwifery students in Ghana. Health Policy Plan.

[11] Mensah-Dapaah J (2012). HIV/AIDS treatment in two Ghanaian hospitals: Experiences of patients, nurses and doctors.

[12] Murira N, Lutzen K, Lindmark G, Christensson K (2003). Communication patterns between health care providers and their clients at an antenatal clinic in Zimbabwe. Health Care Women Int.

[13] World Health Organisation (2014). WHO Statement: The prevention and elimination of disrespect and abuse during facility-based childbirth.

[14] Moyer C, Adongo P, Aborigo R, Hodgson A, Engmann C (2014). ‘They treat you like you are not a human being’: maltreatment during labour and delivery in rural northern Ghana. Midwifery.

[15] Miller S, Lalonde A (2015). The global epidemic of abuse and disrespect during childbirth: hisotry, evidence, interventions and FIGO’s mother-baby friendly birthing facilities initiative. Int J Gynaecol Obstet.

[16] Mannava P, Durrant K, Fisher J, Chersich M, Luchters S (2015). Attitudes and behaviours of maternal health care providers in interactions with clients: a systematic review. Glob Health.

[17] Kruk M, S K, Mbaruku G, K R, Moyo W, Freedman LP (2014). Disrespectful and abusive treatment during facility delivery in Tanzania: a facility and community survey. Health Policy Planning.

[18] Bohren M, Vogel J, Hunter E, Lutsiv O, Makh S, Souza J, Aguiar C, Coneglian F, Araújo Diniz A, Tunçalp Ö (2015). The mistreatment of women during childbirth in health facilities globally: a mixed-methods systematic review. PLoS Med.

[19] McMahon S, George A, Chebet J, Mosha I, Mpembeni R, Winch P (2014). Experiences of and responses to disrespectful maternal care and abuse during childbirth; a qualitative study with women and men in Morogoro region, Tanzania. BMC Pregnancy Childbirth.

[20] Engmann C, Crissman H, Engmann C, Adanu R, Nimako D, Crespo K, Moyer C (2013). Shifting norms: pregnant women’s perspectives on skilled birth attendance and facility based delivery in rural Ghana. Afr J Reprod Health.

[21] Ith P, Dawson A, Homer C (2013). Women’s perspective of maternity care in Cambodia. Women Birth.

[22] Bohren M, Hunter E, Munthe-Kaas M, Souza JP, Vogel J, Gülmezoglu A (2014). Facilitators and barriers ti facility-based delivery in low and-income countries: a qualitative evidence synthesis. Reprod Health.

[23] Roberts J, Sealy D, Marshak H, Manda TL, Gleason P, Mataya R (2015). The patient-provider relationship and antenatal care uptake at two referral hospitals in Malawi: a qualitative study. Malawi Med J.

[24] Asuquo E, Etuk S, Duke F (2000). Staff attitude as barrier to the utilisation of university of Calabar Teaching Hospital for obstetric Care. Afr J Reprod Health.

[25] Raj A, Dey A, Boyce S, Seth A, Bora S, Chandurkar D, Hay K, Singh K, Das A, Chakraverty A (2017). Associations between mistreatment by a provider during childbirth and maternal health complications in Uttar Pradesh, India. Matern Child Health J.

[26] Barry C, Bradley C, Britten N, Stevenson F, Barber N (2000). Patients’ unvoiced agendas in general practice consultations: qualitative study. Br Med J.

[27] Singer M, Baer H (1987). Cure, care and control: an Ectopic encounter with biomedical obstetrics. Encounters with biomedicine: case studies in medical anthropology.

[28] Martin E (2001). The woman in the body: a cultural analysis of reproduction.

[29] Lazarus E (1988). Theoretical considerations for the study of the doctor-patient relationship: implications of a perinatal study. Med Anthropol Q.

[30] Pappas G (1990). Some implications for the study of the doctor-patient interaction: power, structure, and agency in the works of Howard Waitzkin and Arthur Kleinman. Soc Sci Med.

[31] Foucault M (1978). The history of sexuality, volume 1: an introduction London: Allan Lane.

[32] Julliard K, Vivar J, Delgado C, Cruz E, Kabak J, Sabers H (2008). What Latina patients don’t tell their doctors: a qualitative study. Ann Fam Med.

[33] Gott M, Hinchliff S (2003). Barriers to seeking treatment for sexual problems in primary care: a qualitative study with older people. Fam Pract.

[34] Ortner S (2006). Anthropology and social theory: culture, power, and the acting subject.

[35] Scott J (1990). Domination and the arts of resistance: hidden transcripts.

[36] Scott J (1985). Weapons of the weak: everyday forms of peasant resistance.

[37] Giddens A (1979). Central roblems in social theory: action, structure, and contradiction in social analysis.

[38] Franz C, Stewart A, Franz C, Stewart A (1994). Introduction: women’s lives and theories. Women Creating Lives: Identities, Resilience & Resistance.

[39] Ahearn L (2001). Language and agency. Annu Rev Anthropol.

[40] Lock M, Kaufert PA (1998). Pragmatic women and body politics.

[41] Goffman E (1959). The presentation of self in everyday life.

[42] Trends in maternal mortality: 1990 to 2015. Estimates by WHO, UNICEF, UNFPA, World Bank Group and the United Nations Population Division. http://www.who.int/gho/maternal_health/countries/gha.pdf. Accessed 17 July 2017.

[43] Ghana Statisical Service, Ghana Health Service, ICF I (2015). Ghana demographic and health survey 2014.

[44] Ofosu-Amaah S (1981). The maternal and childbirth cervices in Ghana. J Trop Med Hyg.

[45] Amponsah NA (2011). Colonizing the womb: women, midwifery, and the state in colonial Ghana.

[46] Ghana health workforce observatory: Human resources for health country profile. http://www.moh.gov.gh/wp-content/uploads/2016/02/Ghana-hrh-country-profile.pdf. Accessed 20 Apr 2017.

[47] Ghana Health Service: 2014 Family health report. Accra. Ghana Health Service; 2014. p. 119.

[48] Ghana Health Service (2017). 2016 Annual Report.

[49] van der Geest S, Finkler K (2004). Hospital ethnography: introduction. Soc Sci Med.

[50] Hammersley M, Atkinson P (1993). Ethnography: principles in practice.

[51] Spradley J (1980). Participant observation.

[52] Wind G (2008). Negotiated interactive observation: doing fieldwork in hospital settings. Anthropol Med.

[53] Emerson R, Fretz R, Shaw L (2011). Writing Ethnographic Field notes.

[54] Strauss A, Corbin J, Denzin N, Lincoln Y (1994). Grounded theory methodology. Handbook of Qualitative Research.

[55] Geertz C (1973). Thick description: Toward an interpretive theory of culture.

[56] Bloor M, McIntosh J, Cunninigham-Burley S, McKeganey N (1990). Surveillance and concealment: a comparison of techniques of client resistance in therapeutic communities and health visiting. Readings in Medical Sociology.

[57] Irwin S, Jordan B (1987). Knowledge, practice, and power: court-ordered cesarean sections. Med Anthropol Q.

[58] Mulemi B (2010). Coping with cancer and adversity: hospital ethnography in Kenya.

[59] Rahmani Z, Brekke M (2013). Antenatal and obstetric care in Afghanistan – a qualitative study among health care receivers and health care providers. BMC Health Serv Res.

[60] Afsana K, Rashid S (2001). The challenges of meeting rural Bangladeshi women’s needs in delivery care. Reprod Health Matters.

[61] Assimeng M (1999). Social structure of Ghana: A study in persistence and change.

[62] van der Geest S (1997). Between respect and reciprocity: managing old age in rural Ghana. Southern African J Gerontol.

[63] Adinkrah M (2012). Better dead than dishonored: masculinity and male suicide behavior in contemporary Ghana. Soc Sci Med.

[64] Zaman S (2005). Broken limbs, broken lives: ethnography of a hospital ward in Bangladesh.

[65] Adongo P, Phillips J, Kajihara B, Fayorsey C, Debpuur C, Binka F (1997). Cultural factors constraining the introduction of family planning among the Kassena-Nankana of Northern Ghana. Soc Sci Med.

[66] Lipsky M (1980). Street-level bureaucracy: dilemmas of the individual in public services.

[67] Aberese-Ako M, van Dijk H, Gerrits T, Arhinful DK, Agyepong IA (2014). ‘Your health our concern, our health whose concern?’: Perceptions of injustice in organizational relationships and processes and frontline health worker motivation in Ghana. Health Policy Plan.

[68] Agyepong IA, Anafi P, Asiamah E, Ansah E, Ashon D, Narh-Dometey C (2004). Health worker (internal customer) satisfaction and motivation in the public sector in Ghana. Int J Health Plann Manag.

