# Supplementary material for: Mothers’ reproductive and medical history misinformation practices as strategies against healthcare providers’ domination and humiliation in maternal care decision-making interactions: an ethnographic study in Southern Ghana
Source: BMC Pregnancy Childbirth. 2018 Jul 3;18:274. doi: 10.1186/s12884-018-1916-9 (PMC6029400; doi:10.1186/s12884-018-1916-9)
Supplement: Supplementary file 1 — Semi-structured observation and conversation guide. Guide used to capture observations and interactions between pregnant women and healthcare providers during care provision. To also capture interactions between healthcare providers concerning mothers’ care and interview with pregnant women. (DOC 80 kb) [file 12884_2018_1916_MOESM1_ESM.doc]

**OBSERVATION AND INTERVIEW GUIDE**

| Mother’s Pseudonym:__________________ | Number in the series of recruited mothers  [ ] |
| --- | --- |
| Facility Pseudonym : __________________ | Age : ______ |
| Gestational Age at recruitment:__________ | Date at 1st ANC Registering ___________ |
| Occupation : ______________ | Educational Level: _______ |
| Place of Residence:  _________________ |  |
| Mother belongs to Consulting room No?______ |  |

Like I informed you earlier, I am undertaking a study in this facility hospital aimed at improving the quality of care mothers and newborns receive. Therefore, your views and opinion of your care experiences, your interactions with the healthcare provider and some practices mothers and pregnant women sometimes engage in when they seek care. The information you provide is for the research purposes, to improve the quality of maternal and newborn care in the hospital and may be the entire country. Any information you provide will not be disclosed to any of the healthcare providers, so feel free with your responses.

**Section A: INITIAL ANC Visit**

1. **Observation of History taking of mother and care interactions with healthcare provider.**

Brief notes of key issues healthcare provider and mother discussed during the ANC history taking, care consultation and management.

Brief notes describing mothers expressions, easiness and level of contribution during care decision-making interaction?

1. **Interview with mother after ANC care consultation**

Madam, can you please tell me your general impressions about care interactions and relations with you during care consultations? Why do you have this opinion?

**Please during the history taking, tell me some of the information you were unable to provide or did not provide accurately to the healthcare provider. I will go through the sections one after the other:**

- Personal History and Social history (such as occupation, marital status, age, number of months of pregnancy at booking)
- What about information on your Obstetric history (number of pregnancy, total number of children alive , number of induced abortion, spontaneous abortion; gestational age)
- What of information on past pregnancies (such as birth spacing, pregnancy losses and still births)
- Information on Medical and Surgical History
- What other information were you unable to provide to the healthcare provider?

Kindly tell me any other crucial information you were unable to provide the during the care decision-making interaction? Why were you unable to give that information? What are your plans of telling them?

Kindly tell me tell me the various reasons that made you not provide all the required or not provide it accurately (including that of my presence)

Please tell me, did you have any worries and concerns about what was discussed in the consulting room? Tell me, why it was a source of worry to you? How would you address it?

Is there anything else you want us to discuss?

**Next ANC appointment date**

**Thank you**

| Observation & Interview Visit 2 | Date Present Not Present Reasons |
| --- | --- |
| Observation & Interview Visit 3 | Date 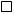 Present Not present 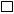 Reasons |
| Observation & Interview Visit 4 | Date 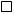 Present Not present 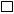 Reasons |
| Observation &Interview Visit 5 | Date 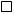 Present Not present 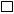 Reasons |
| Observation & Interview Visit 6 | Date 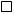 Present Not present 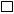 Reasons |
| Observation & Interview Visit 7 | Date 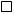 Present Not present 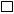 Reasons |
| Observation & Interview Visit 8 | Date 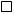 Present Not present 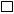 Reasons |
| Observation & Interview Visit 9 | Date 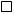Present Not present 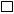 Reasons |
| Observation & Interview Visit 10 | Date 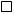 Present Not Present 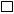 Reasons |
| Observation & Interview Visit 11 | Date 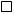Present Not Present 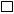 Reasons |

**SUBSEQUENT ANC VISITS**

1. **Observation of Mothers’ Care interactions and consultation with Healthcare provider**

Brief notes of key issues healthcare provider and mother discussed in the ANC consultation, per visit

Brief notes describing mothers expressions, easiness and level of contribution during care decision-making interaction and contribution in the process?

Brief notes describing care givens and key issues discussed between provider and clients? What was your personal impression of the process?

1. **Interview with mother on subsequent ANC care consultation**

Please tell how you are feeling today?

Can you please tell me your general impressions about care interactions and the healthcare provider relations with you during care consultations? Why do you have this opinion?

Please tell me any worries and concerns you had about what was discussed? Why it is the source of worry and how you would you address it?

Did you understand all the management you received? What about what was communicated during the care decision-making interactions?

Was there any aspects of the care management and instruction that were unclear? Kindly tell me which aspects were unclear and how do you intend to address it?

What are your intention to follow the instructions given you by the healthcare providers? Why would you follow them and why not ?

Kindly tell me any crucial information you were unable to provide the healthcare provider during the care interaction? Why were you unable to give that information?

Are there some concerns you would like to share with me about the care decision interaction and management given?

What were some of the good things or impressive you have about the interactions and the consultations you want share with me? Why do you view them as impressive?

Is there anything else you want us to discuss?

**Document Next scheduled ANC appointment**

**Thank you**

**Labour Ward**

| Mothers Pseudonym:__________________ | Number in the series of recruited mothers [ ] |
| --- | --- |
| Date admitted into the labour ward | Date of delivery |
| Type of delivery  Spontaneous  Assisted Vagina Delivery, reasons for this form of delivery  C/s reasons for this form of delivery  Referred:  Reasons for referral outside the hospital |  |

Briefly describe and document how the delivery went?

Briefly describe the birth outcome and conditions of mother and baby

Briefly describe and document any complications mother and baby developed after delivery and how they were managed?

Briefly describe and document any complications mother developed during or after delivery healthcare provider(s) mention or discussed was related information to issues not given or inappropriately given by mother.

POSTNATAL

Day mother and baby arrived for First PNC

PNC Day 48 hours
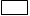
 PNC Day Seven
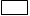
 Six Weeks PNC

1. Brief notes describing care givens and key issues discussed between provider and clients? What was your personal impression of the process?
2. Discussions with Mother after receiving care

Congratulations

Please tell how are you and the baby feeling today?

Can you please tell me your general impressions about care received during and after labour?

What were some of the moments you viewed as impressive you want share with me? Why do you view it as impressive?

Did you have any concerns you want to share with me? Why are they a concern to you?

Thank you
