# Supplementary material for: Mothers’ reproductive and medical history misinformation practices as strategies against healthcare providers’ domination and humiliation in maternal care decision-making interactions: an ethnographic study in Southern Ghana
Source: BMC Pregnancy Childbirth. 2018 Jul 3;18:274. doi: 10.1186/s12884-018-1916-9 (PMC6029400; doi:10.1186/s12884-018-1916-9)
Supplement: Supplementary file 2 — Focus Group Discussion Guide (FGD guide). A topic guide used to explore in-depth pregnant women reproductive and medical misinformation practices among pregnant women and postnatal mothers. (DOC 64 kb) [file 12884_2018_1916_MOESM2_ESM.doc]

**FOCUS GROUP DISCUSSION GUIDE-MOTHERS**

Facility Pseudonym : _____________________

Type of Group: ANC PNC

Discussion Date: (ddmmmyyyy) |_____|_____|____|_____| ____| ____|_____|_____|

Discussion Venue ________

Time started (GMT):___:___

□ Introduce facilitator and any team member with you.

My Name is ……………………………………………………. I am a student and pursing a course in medical Anthropology. This woman with me who is called ........................................... She is a colleague and a worker another part of the region. She is assisting me gather information from pregnant women and lactating mothers who assess healthcare from you facility. Like I informed you earlier, I am undertaking a study in this hospital and it is aimed at improving quality of care mothers and newborns care. You have been selected to be part of this discussion because you are accessed maternal care from this hospital and agreed to be part of the discussions. The discussions will solicit your views and care experiences and some practices mothers and pregnant women engage, when you seek care maternal and newborn care services. My colleagues would ask you a set of questions and I will write the information you provide. The information will also be tape-recorded as a backup for the notes I write because usually this form of discussions tend go faster than the note-taker can write.

Before, we begin she will take you through an informed consent process, a process which is strictly in accordance with the ethical principles governing this research. She would also inform us about the ground rules for the discussions.

□ Please go through consent process, before setting the FGD ground rules

□ **Ground rules:** As she has explained, I will highlight all the items on the informed consent. When I am done, I will give everyone two copies of the form to either sign or thump print. You will keep a copy and we will also keep a copy.

The conversation will be recorded on a tape recorder and the words spoken will be typed into a computer. This will help us to compare your experiences with that of women in another in hospital where my researcher is undertaking a similar study, as well as those of women in other parts of Ghana and in the world. For this reason, it is important to have one person speak at a time, in order to hear everyone’s ideas and opinions.

□ There are no right or wrong answers to the questions we ask, we only want your ideas, experiences and opinions which are valuable and important to hear.

□ Please do not mention your name, names of midwives, nurses or doctors and your hospital when making a contribution. You can say the doctor, nurse or “my hospital” or another hospital I attended elsewhere, to keep all information you provide confidential.

□ We also encourage you that all information we discuss should stays in this room and discussed elsewhere after the FGD.

□ Please do you have any questions to ask?

□ Please, document Discussion START TIME and begin discussion

| **Domain** | | **Topic and probes** | |
| --- | --- | --- | --- |
| **Maternal and Newborn health seeking behaviour** | | **Please Let’s talk about maternal and new-born health seeking care practices.**   - Kindly tell me where women in your community normally seek care for: - Pregnancy services and related issues - Labour and delivery - Postnatal - Newborns - Where did you access care for your last pregnancy or this current pregnancy - Did this place changed in the course of time? Kindly tell me why you changed hospitals? | |
| **Women’s concerns, fears worries in pregnancy and ways of addressing them** | | **Can we please discuss some concerns and worries pregnant women we have in during pregnancy and after pregnancy and how they deal with it?**   - Kindly tell us some of the fears, concerns and worries you or other women you know have when pregnant or have delivered? Why do you or they have these concerns? - How do you or other women normally address the concerns? Why do you take this approach? - What can our midwives and doctors do to help us overcome these concerns and worries? | |
| **Mothers expectation of care provider and influences on mothers satisfaction** | | **Our next discussion will be on our expectations of our healthcare providers during care decision-making interaction and care encounter as a whole**   - Kindly tell us, what do you expect from our healthcare provider when we interact with them during the care decision-making? Why do you have this expectations? What about the entire care encounter? - Tell us how these expectations were or are being met during our care decision-making interactions with our healthcare provider, and the care encounter generally? If these were not, how did you feel and dealt with it? | |
| **Mothers poor adherence to care advice and factors influencing this decision and implication on quality maternal care** | **Please we will next discuss mothers decision to poorly adhere to healthcare providers management advise and factors influencing it**   - We have found that sometimes mothers do not follow all the instructions given them by their healthcare provider, kindly tell us reasons pregnant women do this and what accounts for these decisions in your view? - Please, sometimes healthcare provider inform some pregnant women they would need C/S (operation) for delivery, in your view how do you think pregnant women take this information? - Sometimes women fail appear on the scheduled CS appointment dates, can you please discuss why this often happens?   Looking out for and prompting when not mentioned; **financial, religious leaders influence, partner influence, religion, cultural, fear, distrust of quality of emergency care** | |  |
| **Mothers decision to withhold or misinform healthcare providers about their reproductive, obstetric and social histories and factors influence mothers decision** | **Our next topic will be on mothers decision to withhold or misinform health care providers about their obstetric, reproductive, gynecological, medical and social histories**   - Please, it has also been observed that sometimes pregnant women do not give the right information about themselves to the doctors and nurses? How true is this in your views? - Can you please tell us all information pregnant women or mother often take out at antenatal or accessing maternal care? How about obstetric, medical and reproductive information, do women withhold or misrepresent this information when they are interacting with their healthcare providers? - Kindly tell us other essential medical condition pregnant women that they misinform or withhold form care healthcare providers? - In your view, how common is the practice? Can you tell us the various reasons that account for this practice? | |  |
|  | - Why would any pregnant woman engage in this practice but at the same time expect quality of care? | |  |
|  | - Please tell us, what can be done to encourage pregnant women and mothers to provide all the information healthcare providers to receive better care? | |  |
| **Mothers views of caregivers interaction and relationship with them and implications on satisfaction** | **Our last question is about your opinions of care givers interaction with you during care decision-making interaction and provision of care?**   - Can you please tell us your general impressions about your care interactions and relationship with healthcare providers during care consultations? Why do you have this opinion? - Please recommendations would you give healthcare providers and this hospital concerning this? Why would you not do so? | |  |
| **Closing** | - Please we have ended our discussion. But please do tell us any questions, comments or suggestions you have? - Summarize key themes/information captured for mothers to make inputs and address information not captured appropriately - Thank discussants for their time - Please document DISSCUSSION END TIME | |  |
